# Aztreonam–avibactam therapy following surgical source control for *bla*_*NDM*_-positive carbapenem-resistant *Klebsiella pneumoniae* intra-abdominal infection in an infant: a case report

**DOI:** 10.3389/fphar.2026.1897083

**Published:** 2026-08-14

**Authors:** Jie Liu, Xiaoli He, Wanlin Zhao, Lijia Du, Lina Qiao, Lu Qing

**Affiliations:** 1 Pediatric Intensive Care Unit, Department of Pediatrics, West China Second University Hospital, Sichuan University, Chengdu, Sichuan, China; 2 Key Laboratory of Birth Defects and Related Diseases of Women and Children (Sichuan University), Ministry of Education, Chengdu, Sichuan, China; 3 NHC Key Laboratory of Chronobiology (Sichuan University), Ministry of Education, Chengdu, Sichuan, China

**Keywords:** aztreonam–avibactam, carbapenem-resistant *Klebsiella pneumoniae*, case report, complicated intra-abdominal infection, infant, New Delhi metallo-β-lactamase

## Abstract

Treating carbapenem-resistant *Klebsiella pneumoniae* (CRKP) is challenging, particularly when resistance is mediated by New Delhi metallo-β-lactamase (NDM). Clinical experience with fixed-combination aztreonam–avibactam (ATM–AVI) in young infants remains extremely limited. Here, we report a case of a 2-month-old boy who underwent emergency laparotomy for intestinal obstruction owing to small bowel volvulus with segmental necrosis, followed by bowel resection and reanastomosis. His postoperative course was complicated by respiratory failure, coagulopathy, septic shock, recurrent abdominal distension, and massive ascites. Blood cultures were negative, whereas metagenomic next-generation sequencing of blood detected *K. pneumoniae* and *Enterococcus faecium*. Owing to the worsening of his condition, diagnostic paracentesis and repeat laparotomy were performed, revealing an anastomotic leak. Intraoperative pus culture yielded *bla*_*NDM*_-positive CRKP. The isolate was resistant to carbapenems and ceftazidime–AVI but was susceptible to ATM–AVI, with a minimum inhibitory concentration of ≤4 mg/L. ATM–AVI was administered at 30 mg/kg every 8 h, based on the aztreonam component, for 14 days; linezolid and fluconazole were co-administered as part of the broader anti-infective regimen. The combination of definitive surgical source control, targeted antimicrobial therapy, and comprehensive intensive care successfully controlled the infection and stabilized his hemodynamics. The patient defervesced within 24 h of the first ATM–AVI dose. Inflammatory markers declined rapidly, allowing safe extubation. Follow-up abdominal cultures remained negative. No hepatic or renal toxicity was observed during treatment. This case suggests that, when combined with adequate surgical source control, ATM–AVI may represent a valuable mechanism- and susceptibility-guided pharmacological component in a multidisciplinary treatment paradigm for severe *bla*_*NDM*_-positive CRKP infections in selected infants.

## 1 Introduction

Carbapenem-resistant *Klebsiella pneumoniae* (CRKP) is a major cause of healthcare-associated infection, with limited treatment options (Hao et al., 2022). Its management is especially challenging when resistance is mediated by New Delhi metallo-β-lactamase (NDM), which hydrolyzes carbapenems and most other β-lactams and is not inhibited by the currently available β-lactamase inhibitors (Wilhelm et al., 2024). Although aztreonam (ATM) remains stable against metallo-β-lactamases, NDM-producing *Enterobacterales* often co-produce serine β-lactamases that can inactivate ATM. Avibactam (AVI) inhibits several of these enzymes, providing a strong mechanistic rationale for fixed-combination aztreonam–avibactam (ATM–AVI) therapy (Giuliano et al., 2026).

Most of the available pediatric evidence involves ceftazidime–avibactam with or without aztreonam, whereas clinical experience with ATM–AVI remains particularly scarce. Studies have largely reported bloodstream infections, pneumonia, mediastinitis, or neonatal sepsis, whereas evidence on the ATM–AVI therapy for postoperative complicated intra-abdominal infection (cIAI) is scarce (Hobson et al., 2019; Coskun and Atici, 2020; Asfour et al., 2022; Nascimento et al., 2023; Bawankule et al., 2023; Mangarov et al., 2023; Cousin et al., 2024; Chen et al., 2024; Alghamdi et al., 2025). In this setting, successful treatment depends on microbiologically active antimicrobial therapy as well as timely and effective source control.

To our knowledge, reports of ATM–AVI for postoperative cIAI caused by *bla*_*NDM*_-positive CRKP in infants are exceedingly rare. Herein, we describe a case of a 2-month-old infant with postoperative cIAI and sepsis caused by *bla*_*NDM*_-positive CRKP who recovered after repeat laparotomy and susceptibility-guided ATM–AVI therapy.

## 2 Case presentation

**FIGURE 1:**
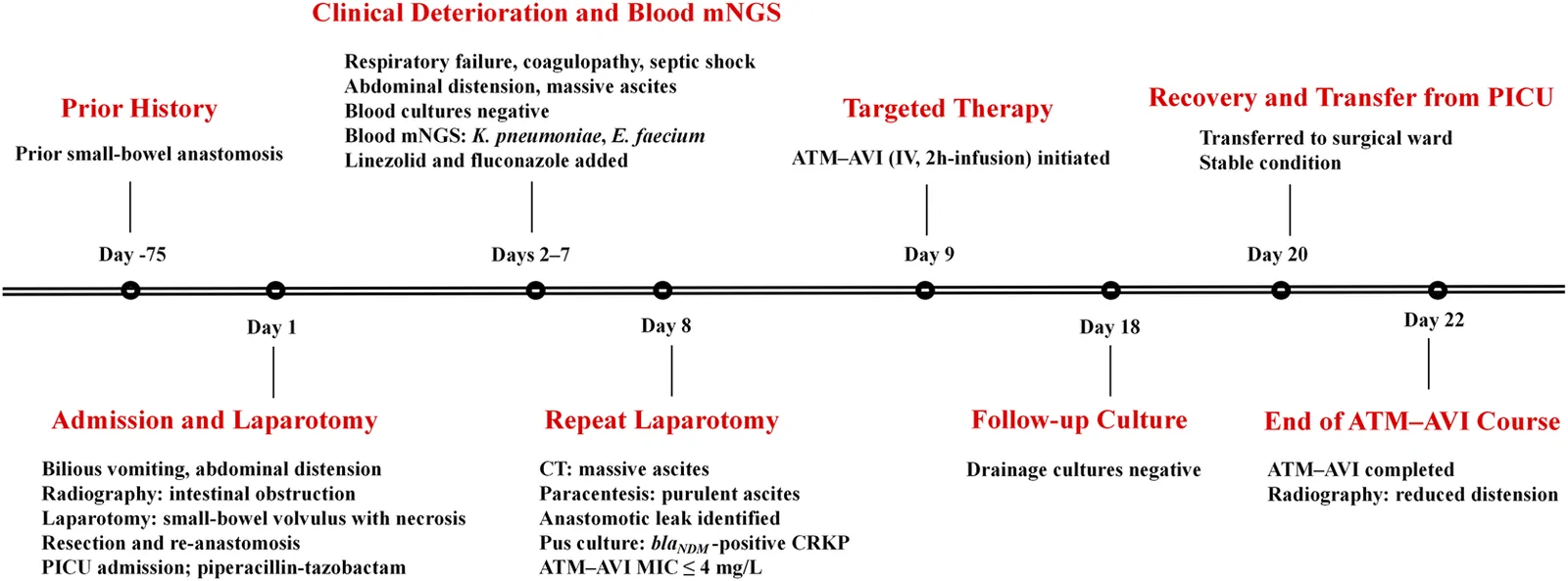
Timeline of clinical deterioration, source control, and targeted ATM–AVI Therapy. Day 1 was defined as the day of admission and initial laparotomy; events before admission are shown as negative days. Abbreviations: PICU, pediatric intensive care unit; mNGS, metagenomic next-generation sequencing; CT, computed tomography; NDM, New Delhi metallo-β-lactamase; CRKP, carbapenem-resistant *Klebsiella pneumoniae*; ATM-AVI, aztreonam–avibactam; MIC, minimum inhibitory concentration; IV, intravenous infusion.

A 2-month-old male infant (born at 35 weeks and 6 days of gestation, with a history of prior small bowel resection and anastomosis performed on day of Life 2) was admitted with bilious vomiting and progressive abdominal distension. Abdominal radiography on admission was suggestive of intestinal obstruction. Emergency laparotomy revealed small bowel volvulus around an adhesive band with segmental bowel necrosis. The necrotic segment was resected, adhesiolysis was performed, and end-to-end anastomosis was completed. The patient was subsequently transferred to the pediatric intensive care unit (PICU), where he received mechanical ventilation. In view of his postoperative status and concern about intra-abdominal infection, empirical piperacillin–tazobactam was initiated; the subsequent clinical course is summarized in Figure 1.

Despite initial postoperative management with piperacillin–tazobactam and organ support, the patient’s subsequent course was marked by progressive clinical deterioration. Respiratory failure, coagulopathy, septic shock, recurrent abdominal distension, and massive ascites occurred during the early postoperative period. Blood cultures remained negative, which may be attributed to prior broad-spectrum antimicrobial exposure as well as the limited pediatric blood sample size (2 mL). However, blood metagenomic next-generation sequencing (mNGS) detected *K. pneumoniae* and *E. faecium,* with 734 and 318 specific reads, respectively, although no resistance gene was identified. *E. faecium* was detected solely by blood mNGS, and no fungal species were isolated from conventional cultures. Nevertheless, given the patient’s septic shock and the high risk of pathogen translocation from the compromised ischemic bowel following prolonged broad-spectrum antibiotic exposure, empirical therapy was promptly initiated. Linezolid was administered to provide systemic Gram-positive coverage, whereas fluconazole was given as empirical prophylaxis against invasive intra-abdominal candidiasis. The initial mNGS results lacked resistance-gene information and susceptibility data, making them inadequate for guiding definitive therapy against *K. pneumoniae*. Thus, source-specific sampling and conventional culture with susceptibility testing were deemed necessary to identify the pathogen and direct targeted treatment.

Despite escalation of antimicrobial therapy and intensive organ support, the patient’s condition continued to deteriorate. Although blood mNGS had identified *K. pneumoniae* and *E. faecium*, the lack of resistance-gene data and rapid clinical decline suggested that an uncontrolled localized postoperative cIAI was more likely than isolated systemic bacteremia. This suspicion was further supported by abdominal computed tomography showing massive ascites; subsequent diagnostic paracentesis yielded purulent fluid. However, the initial aerobic culture was negative, possibly because of prior antibiotic exposure or sampling limitations. Given the patient’s persistent septic shock and the high probability of a localized infectious source, an emergency reoperation was performed on day 8. Intraoperatively, a leak at the recent small-bowel anastomosis was identified, and the affected bowel segment was resected and re-anastomosed to achieve adequate source control.

On day 9, microbiological testing of the intraoperative pus identified CRKP, with polymerase chain reaction confirming the presence of *bla*_*NDM*_. As detailed in the Supplementary Table S1, the isolate exhibited high-level resistance to carbapenems and ceftazidime–AVI but was susceptible to ATM–AVI, with a minimum inhibitory concentration (MIC) of ≤4 mg/L (determined by broth microdilution), interpreted according to the European Committee on Antimicrobial Susceptibility Testing (EUCAST) breakpoints (version 14.0, 2024). Although also susceptible to amikacin, polymyxin B, tigecycline, and levofloxacin, these agents were excluded because of severe pediatric safety concerns, including nephrotoxic and neurotoxic risks, potential cartilage toxicity, and unfavorable pharmacokinetics in systemic sepsis.

Given the presence of persistent septic shock and NDM-mediated resistance, a multidisciplinary consultation, involving pediatric infectious diseases, clinical pharmacology, and intensive care specialists, concluded that ATM–AVI was the safest mechanism-guided treatment. ATM–AVI (Emblaveo; 2 g aztreonam/1 g avibactam per vial; Pfizer) was administered at 30 mg/kg every 8 h, based on the aztreonam component, via 2-h intravenous infusion for 14 days. Owing to the fixed 2:1 aztreonam-to-avibactam ratio, this regimen corresponded to approximately 15 mg/kg of avibactam every 8 h. This off-label regimen was extrapolated from standard pediatric aztreonam guidelines. An 8-h interval was selected to account for the infant’s developmental renal immaturity. To optimize time-dependent pharmacodynamics to maximize the time during which the unbound drug concentration exceeded the MIC (fT > MIC), a prolonged 2-h infusion was implemented with the intention to optimize pharmacokinetic/pharmacodynamic (PK/PD) target attainment. Concomitant linezolid and fluconazole were continued as part of the comprehensive anti-infective strategy.

Clinical stabilization was primarily achieved through a repeat laparotomy on day 8, which resulted in definitive source control and immediately reduced vasopressor requirements. Subsequent ATM–AVI initiation on day 9 controlled the residual infection, with defervescence occurring within 24 h of the first dose. Thereafter, abdominal drainage volume and inflammatory markers progressively declined, allowing safe extubation on day 14. Supplementary Figure S1 details the trends of these markers during treatment. Follow-up abdominal drainage cultures remained negative. Concomitant linezolid was administered for 10 days; this duration was guided by the patient’s progressive clinical stabilization, the continuous decline in inflammatory markers, and negative follow-up drainage cultures. Given the risk of linezolid-induced thrombocytopenia in an infant with baseline coagulopathy, complete blood counts were conducted every 48 h. Platelet counts progressively normalized as septic shock resolved, confirming no exacerbation of myelosuppression. Furthermore, no clinically significant drug-related adverse events were observed, and serial monitoring showed no evidence of hepatic or renal toxicity.

The patient tolerated the transition to enteral nutrition without significant abdominal distension. He was transferred to the surgical ward on day 20 in stable condition and completed the 14-day ATM–AVI course on day 22. At the 3-month outpatient follow-up, the infant demonstrated appropriate growth trajectories, had normal hepatic and renal function panels, and there were no clinical or ultrasonographic signs of recurrent intra-abdominal pathology.

## 3 Discussion

This case illustrates the clinical and pharmacological complexities of treating postoperative cIAI caused by *bla*_*NDM*_-positive CRKP in a young infant. The favorable outcome highlights the synergistic efficacy of a multidisciplinary approach entailing definitive surgical source control, targeted mechanism-guided antimicrobial therapy with ATM–AVI, empirical coverage for concurrent pathogens with linezolid and fluconazole, and intensive supportive care. Here, treatment selection was dictated not only by disease severity and limited alternatives, but also by precise resistance mechanism and *in vitro* susceptibility profile.

The rationale for ATM–AVI in NDM-producing isolates is well established. NDM is a metallo-β-lactamase that hydrolyzes most β-lactams, such as carbapenems, but does not efficiently hydrolyze ATM. However, ATM alone may still be ineffective because NDM-producing *Enterobacterales* often co-produce serine β-lactamases that hydrolyze aztreonam. AVI does not inhibit NDM itself but inhibits many accompanying serine β-lactamases, thereby protecting ATM and restoring activity in susceptible isolates (Montero et al., 2025a; Montero et al., 2025b). In our case, the isolate was resistant to carbapenems and ceftazidime–AVI but was susceptible to ATM–AVI, a pattern consistent with this mechanism. This supports the value of mechanism- and susceptibility-guided therapy in severe multidrug-resistant gram-negative infection.

Clinical experience with ATM–AVI in early infancy remains highly restricted (Roye-Azar et al., 2026). It is approved by the European Medicines Agency for certain infections in adults but has no US Food and Drug Administration approval or pediatric indications (Al Musawa et al., 2024). For our patient, therapeutic options were restricted. First, ceftazidime–AVI alone lacks activity against NDM-producing organisms, carbapenems were ineffective, and aminoglycosides or polymyxins raise major toxicity concerns in young infants (Ngiam et al., 2026). Second, fluoroquinolones are associated with risk of severe musculoskeletal toxicity (Jackson et al., 2016). Third, tigecycline is limited by its rapid extravascular distribution, making it pharmacokinetically inadequate for resolving concurrent bacteremia (Tasina et al., 2011). Most pediatric studies have reported bloodstream infection, pneumonia, mediastinitis, or neonatal sepsis, and several have used ATM combined with ceftazidime–AVI rather than ATM–AVI product (Hobson et al., 2019; Coskun and Atici, 2020; Asfour et al., 2022; Nascimento et al., 2023; Bawankule et al., 2023; Mangarov et al., 2023; Cousin et al., 2024; Chen et al., 2024; Alghamdi et al., 2025) (Table 1). Evidence for fixed-combination ATM–AVI therapy in postoperative cIAI in infants is particularly limited, making this case clinically relevant.

**TABLE 1:** Published pediatric reports of ceftazidime–avibactam, aztreonam, and/or aztreonam–avibactam therapy for carbapenem-resistant gram-negative infections.

Study	Age	Underlying conditions	Pathogen and resistance mechanism	Infection type	Regimen	CAZ–AVI and/or aztreonam-based duration	Outcome
Hobson et al. (2019)	3 years	Relapsed acute lymphoblastic leukemia, aplasia, mucositis	*Morganella morganii*, NDM-1	Bacteremia	Aztreonam + CAZ–AVI	10 days	Clinical cure; no relapse at 6 months
Coskun and Atici (2020)	27 weeks’ gestation	Prematurity, respiratory distress syndrome, necrotizing enterocolitis	CRKP	Urinary tract infection	CAZ–AVI	10 days	Clinical cure
Asfour et al. (2022)	27 weeks’ gestation	Prematurity, respiratory distress	*K. pneumoniae*/CRE, MRSE, vancomycin-resistant *enterococci*	Meningitis and bacteremia	CAZ–AVI + colistin + linezolid	21 days	Clinical improvement, extubated, discharged at CGA 42+5 weeks
Asfour et al. (2022)	28 weeks’ gestation	Prematurity, respiratory distress	MRSE*,* CRKP	Bacteremia	CAZ–AVI + amikacin	5 days	Died on day 61
Nascimento et al. (2023)	29 weeks’ gestation	Prematurity, respiratory distress syndrome	*Staphylococcus haemolyticus*; CRKP	Bacteremia, infectious ileum, urinary tract infection	CAZ–AVI + ciprofloxacin	14 days	Clinical cure; discharged on day 109
Bawankule et al. (2023)	27 weeks’ gestation, day 9 of life	Hyaline membrane disease, patent ductus arteriosus, and suspected necrotizing enterocolitis	CRKP, NDM plus OXA-48-like	Bacteremia	Cefiderocol + polymyxin B+ CAZ–AVI + aztreonam	14 days	Clinical cure; microbiological clearance; well at 1 year
Mangarov et al. (2023)	24 days	Prematurity, low birth weight	Pan-drug-resistant *K. pneumoniae ss.*	Bacteremia; suspected gastrointestinal infection	CAZ–AVI + imipenem/cilastatin + metronidazole + vancomycin	17 days	Clinical cure
Cousin et al. (2024)	13 months	DiGeorge syndrome, cardiac surgery	*E. coli*, NDM-1	Mediastinitis	Aztreonam + CAZ–AVI + fosfomycin	44 days	Clinical cure; no relapse at 10 weeks
Chen et al. (2024)	32 weeks’ gestation	Prematurity, very low birth weight, neonatal respiratory distress syndrome, necrotizing enterocolitis, and surgery	*K. pneumoniae*, OXA-48 plus ESBL	Bacteremia	CAZ–AVI + aztreonam + fosfomycin + fluconazole	Approximately 30 days	Clinical cure; discharged on day 58
Alghamdi et al. (2025)	30 weeks’ gestation	Prematurity, respiratory distress syndrome	MDR *S. maltophilia* plus *B. cepacia,* MRSA and *E*. *faecium*	Respiratory infection, bacteremia	Aztreonam + CAZ–AVI + teicoplanin + trimethoprim–sulfamethoxazole	14 days	Microbiological clearance; extubated
Present case	2 months	Prior bowel surgery, anastomotic leak	Intraoperative pus: *bla*_*NDM*_ *-*positive CRKP; blood mNGS: *E. faecium* also detected	Postoperative complicated intra-abdominal infection with sepsis	ATM–AVI + linezolid + fluconazole	14 days	Clinical improvement; afebrile within 24 h; microbiological clearance; extubated; transferred to surgical ward

Abbreviations: CAZ–AVI, ceftazidime–avibactam; NDM, New Delhi metallo-β-lactamase; CRKP, carbapenem-resistant *K. pneumoniae*; CRE, carbapenem-resistant *Enterobacterales*; MRSE, methicillin-resistant *Staphylococcus epidermidis*; CGA, corrected gestational age; OXA, oxacillinase; ESBL, extended-spectrum β-lactamase; MDR, multidrug-resistant; MRSA, methicillin-resistant *Staphylococcus aureus*; mNGS, metagenomic next-generation sequencing; ATM–AVI, aztreonam–avibactam.

Given the virtual absence of pharmacokinetic data for ATM–AVI in infants younger than 3 months (Franzese et al., 2022; Castro-Frontiñán et al., 2025; Bradley et al., 2025), dose selection required rigorous empirical extrapolation based on established PK/PD principles. For β-lactams, the primary determinant of efficacy is fT > MIC (Al Musawa et al., 2024; Craig, 2003). Accordingly, we based our regimen on standard pediatric aztreonam dosing (30 mg/kg/dose) and selected an 8-h interval to accommodate developmental renal immaturity, preventing drug accumulation while maintaining therapeutic serum levels. The strategic use of a prolonged 2-h infusion was intended to maximize fT > MIC, aiming to optimize drug exposure and tissue penetration into the peritoneal biophase, which is often compromised by postoperative capillary leak and hemodynamic instability (Yalcin et al., 2022; Severino et al., 2023; Raber et al., 2026). The favorable response supports the feasibility of this regimen, though further pediatric PK/PD studies are imperative to define optimal exposure targets.

While optimizing the PK/PD index of ATM–AVI is critical, adequate drug penetration into the peritoneal cavity requires resolving local anatomical barriers. The anastomotic leak acted as a continuous source of bacterial inoculation, driving the persistent sepsis. Therefore, the repeat laparotomy was a critical prerequisite for clinical reversal. This case reinforces a paramount principle in cIAI management, where even the most potent antimicrobial regimen will fail if the primary infectious focus remains physically unaddressed.

This case also highlights the importance of integrating different diagnostic approaches. Blood mNGS suggested systemic infection, but did not provide enough information for definitive targeted treatment. The decisive microbiological result was derived from intraoperative pus culture, which identified *bla*_*NDM*_-positive CRKP and enabled susceptibility-guided use of ATM–AVI. In severe multidrug-resistant infection, source-specific culture and susceptibility testing are essential, whenever feasible (Sartelli et al., 2021; Sartelli et al., 2025).

This study has several limitations. First, as a single-case report, attributing the patient’s clinical recovery solely to ATM–AVI oversimplifies the pathophysiology. Intestinal barrier disruption likely facilitated translocation of enteric commensals, exacerbating shock. While CRKP was isolated from the primary pus specimen, blood mNGS detected *E. faecium*. Furthermore, it must be acknowledged that mNGS detects microbial DNA rather than viable organisms; thus, positive findings require careful interpretation within the overall clinical context. Nevertheless, given the patient’s critical condition, empirical coverage of this uncultured pathogen with concurrent linezolid likely played a parallel role in stabilizing hemodynamics. Therefore, ATM–AVI functioned as a key targeted therapy against the primary source within an essential broader combination regimen. Second, the lack of therapeutic drug monitoring for ATM–AVI in this critically ill infant limited our ability to objectively verify pharmacokinetic target attainment. Consequently, the adequacy of drug exposure could only be inferred from empirical PK/PD principles and the observed clinical response.

## 4 Conclusion

This report presents the case of a 2-month-old infant with postoperative cIAI and sepsis caused by *bla*_*NDM*_-positive CRKP who recovered following multidisciplinary intervention. This case supports the potential role of ATM–AVI as a mechanism- and susceptibility-guided option for severe NDM-producing *Enterobacterales* infections in selected infants. However, favorable outcomes remain fundamentally dependent on definitive surgical source control and comprehensive intensive care. Further pediatric studies are urgently needed to define the pharmacokinetics, safety, and optimal dosing of ATM–AVI in early infancy.

## Data Availability

The raw data supporting the conclusions of this article will be made available by the authors, without undue reservation.
