# Supplementary material for: Aztreonam–avibactam therapy following surgical source control for bla NDM -positive carbapenem-resistant Klebsiella pneumoniae intra-abdominal infection in an infant: a case report
Source: Front Pharmacol. 2026 Aug 14;17:1897083. doi: 10.3389/fphar.2026.1897083 (PMC13522163; doi:10.3389/fphar.2026.1897083)
Supplement: Supplementary file 1 [file Table1.docx]

**Supplementary Table S1** Comparison of antimicrobial susceptibility results between peritoneal drainage fluid and intraoperative pus specimens from the same patient

| Antibiotics | Drainage Fluid (scanty growth) | Result | Intraoperative Pus (moderate growth) | Result | Consistency |
| --- | --- | --- | --- | --- | --- |
|  | MIC (mg/L)/ Zone (mm) |  | MIC (mg/L)/ Zone (mm) |  |  |
| Aminoglycosides | | | | |  |
| Amikacin | ≤ 2.0 | S | ≤ 2.0 | S | Consistent |
| Gentamicin | 17 (mm) | I | 18 (mm) | I | ^*^Concordant |
| β-lactams | | | | |  |
| Aztreonam | 27 (mm) | S | 29 (mm) | S | Consistent |
| Aztreonam-avibactam | 29 (mm) | S | 30 (mm) | S | Consistent |
| Amoxicillin-clavulanate | ≥ 32.0 | R | ≥ 32.0 | R | Consistent |
| Ampicillin | 6 (mm) | R | 6 (mm) | R | Consistent |
| Cefazolin | 6 (mm) | R | 6 (mm) | R | Consistent |
| Cefepime | ≥ 32.0 | R | ≥ 32.0 | R | Consistent |
| Cefoperazone-sulbactam | ≥ 64.0 | R | ≥ 64.0 | R | Consistent |
| Ceftriaxone | ≥ 64.0 | R | ≥ 64.0 | R | Consistent |
| Cefoxitin | ≥ 64.0 | R | ≥ 64.0 | R | Consistent |
| Ceftazidime | ≥ 64.0 | R | ≥ 64.0 | R | Consistent |
| Cefuroxime | ≥ 64.0 | R | ≥ 64.0 | R | Consistent |
| Piperacillin-tazobactam | ≥ 128.0 | R | ≥ 128.0 | R | Consistent |
| Carbapenems | | | | |  |
| Ertapenem | ≥ 8.0 | R | ≥ 8.0 | R | Consistent |
| Imipenem | ≥ 16.0 | R | ≥ 16.0 | R | Consistent |
| Meropenem | 10 (mm) | R | 10 (mm) | R | Consistent |
| Fluoroquinolones | | | | |  |
| Levofloxacin | ≤ 0.12 | S | ≤ 0.12 | S | Consistent |
| Folate pathway inhibitors | | | | |  |
| Trimethoprim-sulfamethoxazole | ≤ 1/19 | S | ≤ 1/19 | S | Consistent |
| Glycylcyclines | | | | |  |
| Tigecycline | ≤ 0.5 | S | ≤ 0.5 | S | Consistent |
| Polymyxins | | | | |  |
| ^#^Polymyxin B | 1 (Etest) | S | 0.5 (Etest) | S | Consistent |

Note: S, susceptible; I, intermediate; R, resistant. All interpretations were based on EUCAST breakpoints (version 14.0) [1]. MIC values are in mg/L; zone diameters are in mm and indicated accordingly.

^*^Categorical interpretations were concordant; minor quantitative variations were observed.

^#^Polymyxin B susceptibility was determined by Etest for both specimens.

**Reference**

[1] The European Committee on Antimicrobial Susceptibility Testing. Breakpoint Tables for Interpretation of MICs and Zone Diameters. Version 14.0, 2024. Available at: https://www.eucast.org/breakpoints/
